# Leaving the City: Counterurbanisation and Internal Return Migration in Sweden

**DOI:** 10.1007/s10680-023-09649-4

**Published:** 2023-03-08

**Authors:** Erika Sandow, Emma Lundholm

**Affiliations:** 1 Centre for Demographic and Ageing Research, Umeå University, Umeå, Sweden; 2 Department of Geography, Umeå University, Umeå, Sweden

**Keywords:** Counterurbanisation, Return migration, Family migration, Intergenerational networks, Competing risk

## Abstract

This paper examines counterurban migration among young families with children in Sweden and the extent to which these moves reflect return migration, recognising the role of family members and family roots at the destination from a life course perspective. Drawing on register data for all young families with children leaving the Swedish metropolitan areas during the years 2003–2013, we analyse the pattern of counterurban moves and explore how the families’ socioeconomic characteristics, childhood origins, and links to family networks are associated with becoming a counterurban mover and choice of destination. The results show that four out of ten counterurban movers are former urban movers who choose to return to their home region. Among them, almost all have family at the destination, indicating that family ties are important for counterurban migration. In general, urban residents with a background outside metropolitan areas are much more likely to become counterurban movers. Families’ previous residential experiences during childhood, particularly in rural areas, are found to be associated with the residential environment they choose to resettle in when leaving the big city. Counterurban movers making a return move are similar to other counterurban movers in relation to employment status, but tend to be better off economically and move longer distances than other counterurban movers.

## Introduction

Recent research indicates that there is renewed interest in out-migration from metropolitan areas in the Nordic countries (e.g. Denmark and Sweden) (Aner, 2016; Sandow & Lundholm, 2020; Hansen & Aner, 2017; Vogiazides & Kawalerowicz, 2022) as well as in the Netherlands (Karsten, 2020). Many of these out-migrants are families with children (Andersen & Nørgaard, 2018; Aner, 2016; Sandow & Lundholm, 2020; Hansen & Aner, 2017; Niedomysl & Amcoff, 2011a). In this paper, the focus is on young families with children who make a counterurban move out of the metropolitan areas in Sweden. When studying these counterurban families, we pay special attention to their family networks and roots to analyse how return migration is interrelated to counterurban migration. While counterurban migration has been portrayed as lifestyle-induced mobility motivated by a search for idyllic rural landscapes, return migration has been seen as a pragmatic and even necessity-driven migration, and as a phenomenon typically separate from counterurbanisation (Barcus & Halfacree, 2017). However, some studies have identified links to extended family members and ‘returning to one’s roots’ as important motives for counterurban migration, especially among young families (e.g. Scott et al., 2017), reflecting how return migration can be part of counterurban migration. By exploring counterurban migration flows directed at childhood areas, this study can contribute to nuancing the understanding of return migration in relation to counterurbanisation and add to a growing body of literature on counterurban migration (Bijker & Haartsen, 2012; Grimsrud, 2011; Karsten, 2020; Stockdale, 2016; Stockdale & Catney, 2014).

Families with children are generally known to be more rooted and less inclined to move compared to younger, and single persons (Bailey et al., 2004; Mulder & Hooimeijer, 1999). While the direction of moves among younger single movers tends to be upward in the urban hierarchy, couples and families typically move in the other direction (Plane et al., 2005). Counterurban migration is important for receiving regions. Families with children moving in as new residents form a group that is vital to many of the smaller and more rural municipalities as they counteract the trend of population ageing, and there are expectations that highly skilled workers can boost the local economy (Bosworth & Atterton, 2012; Roberts & Townsend, 2016).

In this paper, we analyse counterurban migration flows from metropolitan areas to small and medium-sized town areas and rural areas among young families in Sweden. The aim is to examine the impact of childhood origin on young family's propensity to make a counterurban move and what type of destination they resettle in. Further, we explore the interrelation between counterurban migration and return migration and compare the characteristics of return migrants to those of other counterurban movers.

This paper advances the research on counterurban migration not only by highlighting the role of return migration, childhood origin, and family networks but also by distinguishing by destination types. Using register data, this study can complement previous qualitative studies on return migration to rural areas (Pedersen, 2018), showing that family and roots are important for understanding counterurban migration (Scott et al., 2017).

## Background

### Counterurbanisation

Counterurbanisation was originally understood as the opposite of urbanisation, a process of deconcentration of population (Berry, 1976). However, the concept is often used to denominate migration movements downward in the urban hierarchy, i.e. counterurbanising migration (for an extended discussion, see Champion, 1998; Mitchell, 2004). Regarding destinations of counterurban migration, there is no uniform definition. Halliday and Coombes (1995) argue that in order to capture a countertrend to urbanisation it is essential to separate counterurban moves from suburban moves nearby metropolitan areas. One model they propose is what they call ‘anti-metropolitan’ migration, meaning the study of migration out from the larger metropolitan regions to non-metropolitan areas. In this paper, we use the concept of counterurbanisation in a way similar to how Halliday and Coombes do, focusing on the counterstream out from metropolitan areas, while acknowledging that destinations can vary from medium-sized towns to remote rural areas.

In contrast to definitions including all migration downward in the urban hierarchy, counterurban migration has often been equated with migration to rural areas and frequently portrayed as a quest for the rural idyll, typically among the urban middle class. Beyond this stereotype, several attempts have been made to illustrate the diversification among counterurban movers by studying the diversity among different rural areas (Bijker et al., 2013) or by dividing movers into categories based on their motives for moving from urban to rural areas. For example, Mitchell (2004) differentiates between exurbanites who keep their links to urban life while relocating to a more rural environment, displaced urbanisers (those who ‘accidently’ move to rural areas for employment reasons), and anti-urbanisers who actively choose a rural lifestyle and cut their bonds to urban life. Halfacree (2008) proposes a model for categorising counterurban movers based on the importance they attach to the rural environment, ranging from ‘back to the land’ movers, who seek to dramatically change their lifestyle, to ‘default’ counterurban movers who move for instrumental reasons and place little or no emphasis on the rural. Grimsrud (2011) identifies a category that is missing in both Halfacree’s and Mitchell’s definitions, namely what she calls ‘rural by belonging’. She finds that as many as 70% of movers to rural areas in Norway state that they moved to a municipality where they or their partner grew up. Bijker and Haartsen (2012) found that many counterurban movers stated instrumental reasons, related to work and social motivations, for moving to rural areas. In their study, they also distinguish between different kinds of rural areas, with more mainstream counterurban motives being more prevalent among movers to more ‘popular’ rural areas and nearness to family being more important among movers to less popular areas.

### Return Migration

While counterurbanisation has traditionally been portrayed as lifestyle-induced migration, return migration is often understood based on a success–failure dichotomy, whereby the decision to return can be a result of failure (Haartsen & Thissen, 2014; Hunt, 2004). Amcoff and Niedomysl (2013) found that unemployment is a trigger for return migration in Sweden, both in general as well as specifically to metropolitan areas. In these terms, failure is often understood as economic failure, for instance unemployment. Divorce can also trigger migration, and as single individuals are more dependent on support outside the household, those who are single or recently divorced are more likely to be forced to live close to their parents for support (Smits, 2010). Return migration could also be seen as failure in the sense of being a ‘move in the wrong direction’ or even ‘downward social mobility’ among young people who have left rural areas for cities, even if their place attachment and identity are strongly related to a rural place (Pedersen, 2018). Pedersen’s (2018) qualitative study from Denmark found that young people who had moved from rural areas often had a preference for rural living (especially for raising children) but were reluctant to move back to their home region as they perceived this as negative.

There are also other perspectives on return migration, especially when it comes to international migration. Ní Laoire (2007) studied international return migration to rural Ireland and emphasized the interrelationships between counterurbanisation discourses and narratives of return migration. Saar and Saar (2020) also found a strong interlinkage between lifestyle migration and return migration in the case of Estonians moving back from the UK. There are also studies that have found that return migration is typically a socially motivated move (Niedomysl & Amcoff, 2011a) and that, besides employment, family members living in the region of origin and ties to the community were important motives for migrating (von Reichert et al., 2014; see also their 2013 article). Based on the findings of complex drivers of return migration and considering the scope of return migration—estimated to make up between a quarter and a third of all internal moves in Sweden, Australia, and Canada (Amcoff & Niedomysl, 2013; Newbold & Bell, 2001)—it is evident that there is more to return migration than failed migration, and it is also relevant to compare return migrants to other counterurban movers.

In this study, we specifically study young families who move from an urban area as intact couples with children and their return migration. There are reasons to assume that this group differs from other potential return migrants, for example ‘boomerangers’. ‘Boomerangers’ are mainly youth who are not yet established on the labour and/or housing market and/or in a family formation and often chose to move back to their parents’ home in response to ‘glitches’ related to work, housing, or relationships (Berngruber, 2015; Mitchell, 2017; Olofsson et al., 2020). Although support from and the company of parents and other family members could be an important element in the decision among the individuals and families in our study, we can assume that they make more of an independent decision to move close to their parents (rather than moving in with them) based on preferences rather than necessity after ‘failing’, or to return to the parental home as a result of a transition between education, jobs, or partnerships.

### Previous Residential Experiences and Residential Environmental Choices

Research has shown that previous residential experiences influence residential environment choices, particularly in the form of return migration (Feijten et al., 2008). Residential experiences may influence the migration choice due to the social connections to family or friends that tie them to their place of origin. The wish to return to one’s birthplace or place of origin has been shown to have a strong impact on residential choice (Blaauboer, 2011), regardless of whether one’s origin is in an urban, suburban, or rural area (Feijten et al., 2008). Awareness of and attitudes towards a residential environment can also influence the probability of returning to a place. They can also contribute to preferences for moving to the same type of residential environment later in life, even if it is not one’s place of origin. As return migration might be the result of a wish to return to the specific place where a person was born or grew up, the move could thus also entail a move to an environment similar to this place (Feijten et al., 2008). For example, studies have found that having lived in a rural area increased the probability of moving to another rural area—not the person’s birthplace (Feijten et al., 2008; van Dam et al., 2002)—while the effects of urban and suburban living are more location-specific (Feijten et al., 2008). On Ireland, the role of rural origin in rural mobility, including counterurbanisation, is discussed by Scott et al. (2017). They found that in an Irish context there is a significant ‘return-to-roots’ movement within the counterurban flow. Among the Irish counterurban movers, about 60% originated from a rural area and were returning to their area of origin or a similar residential environment, suggesting a flow of return migrants rather than stereotypical urban-to-rural migrants. Scott et al. (2017) as well as other researchers (Stockdale, 2014) suggest that the view of counterurbanisation should be more nuanced, beyond the stereotypical view of urban-dwellers seeking greenspace and rurality. Based on previous research, it can thus be expected that previous residential experiences influence the residential choices of Swedish families leaving the metropolitan areas.

### Linked Lives and Migration

Return migration can also be understood from a life course perspective, acknowledging the linked lives of the household members but also extended to include a relational perspective whereby the household is connected to a network of parents, family, and friends (Elder, 1994). The provision of instrumental support from the older generation to adult children has been found to be substantial, not least in the Scandinavian context (Lestari et al., 2020). The impact of family ties outside the household has often been ignored in migration research (Mulder, 2018), but in recent years researchers have increasingly advocated for a life course perspective on migration whereby the importance of linked lives and family ties for understanding migration is acknowledged (Coulter et al., 2016; Thomas & Dommermuth, 2020).

Return migration is more likely in some parts of the life course than others, typically the family formation phase. In a Swiss study (Rérat, 2014), it was found that young people who had moved from a rural area to study were more likely to return to their origin after graduation if they had children, if their partner was from the same region, or if their parents had originated from the same region. The social connections to a place include not only parents but also siblings and other relatives as well as friends. The role of siblings as migration attractions has been studied by Mulder et al., (2020a, 2020b). However, parents seem to be the main attractor (von Reichert et al., 2013; Zorlu & Kooiman, 2019), and Zorlu and Kooiman (2019) found that if one’s parents no longer live in a region, return migration is much less likely.

Increasingly, studies are also showing that moving closer to family networks is an important motive for counterurban migration (Haartsen & Thissen, 2014; Scott et al., 2017). The counterurbanisers, especially young families, are swapping friends for family (Haartsen & Thissen, 2014; Scott et al., 2017). Recent research also reveals that families with young children are overrepresented in counterurban migration flows (Andersen & Nørgaard, 2018; Sandow & Lundholm, 2020; Hansen & Aner, 2017; Stockdale & Catney, 2014). As discussed by, e.g. Feijten et al. (2008) and Scott et al. (2017), the role of family members and family roots at the destination, associated with return migration, relates to the life course perspective on migration choices. In relation to this, we seek to add to the literature the importance of family networks at the destination, in relation to return migration, for understanding contemporary counterurbanisation in a Swedish context.

### Counterurbanisation and Return Migration in Sweden

Compared to internal migration in general, which in Sweden is dominated by young people (Statistics Sweden, 2018), counterurban movers have a slightly older age profile (Lindgren, 2003). During the 1970s, the out-migration from big cities in Sweden increased and was mainly directed at villages near the cities. Longer counterurban moves directed towards the more remote countryside were few, but increased somewhat during the 1980s. The following period, from the mid-1980s to the mid-1990s, was characterised by continuous urbanisation and suburbanisation rather than counterurbanisation (Lindgren, 2003; Westlund, 2002). Lindgren’s (2003) study of out-migration from major Swedish cities downward in the urban hierarchy from 1985 to 1995 found that counterurban movers were more likely to be less well off, have a university education, and be outside the labour force close to the migration event. Lindgren’s study also showed that the counterurban movers were often single and older, while families with children more seldom made such a move. Since the beginning of the 2000s, the migration flow out of the major cities in Sweden has started to change. Population statistics (Statistics Sweden, 2021) reveal that this out-migration from the metropolitan areas is a persisting trend that accelerated during the first year of the Covid19 pandemic (Vogiazides & Kawalerowicz, 2022). These moves are dominated by young families in the age range of 25 to 40 (Statistics Sweden, 2021). A recent study (Sandow & Lundholm, 2020) shows that from 2003 to 2013 there was a small, but steady, flow of young families with children making a counterurban move from the Swedish metropolitan areas and resettling beyond the suburban landscape. In contrast to the traditional view of counterurbanisation, rural areas are not the main destination choice for the majority of families leaving metropolitan cities; the migration stream out of the Swedish metropolitan areas is directed mainly towards medium-sized or small towns. These families are often highly educated and in employment to a greater extent than those families who choose to stay in the metropolitan area (Sandow & Lundholm, 2020). This growing group—young families with children—is in focus in this study, in which we analyse the extent to which return migration is part of this contemporary counterurban migration phenomenon, as well as the importance of intergenerational networks at the destination.

Previous studies on return migration in Sweden examining the scope of the phenomenon have looked at specific age groups, such as those of retirement age (Lundholm, 2012) or young adults (Mulder et al., 2020a). In a study based on survey data including all internal moves (> 20 km), it was found that 31% stated that they had returned to where they (or their partner) grew up (Niedomysl & Amcoff, 2011b). In the same study, it was also found that social relationships, with proximity to family and friends, serve as an important motivation for moving among this group. Gillespie et al. (2021) used the same survey data to compare return migrants to onward migrants, finding that family was more important in the younger age groups while the importance of friends increased with age. Further, no association was found between return migration and degree of urbanisation, indicating that family and friends are important in all geographical contexts.

When Amcoff and Niedomysl (2013) studied all return migration between regions in Sweden, using register data, they found that the presence of children is a factor that triggers return migration out from metropolitan areas. When studying cohabiting partners in Sweden making any interregional move during 2006, using register data, Amcoff and Niedomysl (2015) found that almost a third were returning to a region of previous residence. Female partners dominated among the returnees, regardless of the type of region of previous residence and whether or not they were economically gaining the most from the return migration. Among partners with children, it was even more likely that the families returned to the mother’s previous residence (Amcoff & Niedomysl, 2015). Recent statistics from population registers reveal that this migration pattern still holds. Almost half of all families with children making a return move within Sweden return to the mother’s birth region, where the father was not born (Statistics Sweden, 2017). This gendered pattern in return migration among families reflects prior migration decisions among young women and men. Young women tend to move more often than men (Lundholm, 2007), particularly in the same year as marriage or childbearing (Mulder & Wagner, 1993), and women move over longer distances at the start of co-residence (Brandén & Haandrikman, 2018). Thus, couples tend to live in closer proximity to the man’s parents than the woman’s parents (Pettersson & Malmberg, 2009). Given the higher migration propensity among young women, this results in a larger number of potential female return migrants. The tendency for women to live further from their parents is the most pronounced in younger ages, but by age 37 the difference is less pronounced, according to a study by Kolk (2017). It can thus be expected that when couples in a later phase of life, after forming a family, decide to make a counterurban move they are more likely to move nearer the female partner’s parents if deciding to resettle closer to family.

Based on previous research on Swedish counterurban moves and return moves, we can assume that family members and family roots at the destination are of importance for young families’ destination choices when moving out from a metropolitan area. Also, from what is known we can expect that family members and family roots are important for all types of destination choices and not only for counterurban moves to rural areas. To our knowledge, there are no studies that take into account the influence of previous residential experiences and closeness to family members on counterurban movers’ destination choices in Sweden. Nor is there recent research on return migration in Sweden, and no studies have previously considered the extent of return migration in Swedish counterurban migration as we do in this study.

## Data and Methods

### Data

Empirically, the study is based on all young families with children living in one of the three Swedish metropolitan areas (Stockholm, Malmö, and Gothenburg) during the period 2003–2013. We focus on the young families in the age 25 to 40 as they dominate the out-migration from the Swedish metropolitan areas (Statistics Sweden, 2021). The families are identified through household registers from Statistics Sweden, with links between partners, parents, and children. Each family has a family identity number (FamId), which is identical to the civil personal number of the eldest of maximum two generations living at the same address. Unmarried cohabiting couples with common children have the same FamId. Cohabiting couples with non-common children do not have the same FamId and are therefore not identified as a family in the data.

The data are longitudinal and contain annual household data on place of residence and work as well as a number of socioeconomic variables (income and education level, age, occupation and country of birth of both partners, number and age of children in the household). We also have information on the municipality of residence at age twelve for both partners (here referred to as place of childhood origin), as well as municipality of residence for both partners’ parents for each year. The longitudinal data allow us to observe families’ previous residential history before they make a move. As the data cover the total population, it is possible to analyse all counterurban moves made between 2003 and 2013, and in combination with the data on childhood residence, return moves within the group of counterurbanising young families can be identified. Here, a counterurban move is defined as a move out of a metropolitan area downward in the urban hierarchy. Return migration is defined here as moving back to the place of childhood origin for one or both of the spouses. The migration is measured as a change in place of residence from one year to another, measured in December of each year. The classification of a metropolitan area and destination used here is based on the Swedish Association of Local Authorities and Regions’ (SKR, 2016) division of municipalities into groups based on structural parameters such as population and commuting patterns. The classification contains three main groups (metropolitan area, medium-sized town area, and rural area), with the municipalities divided into a total of nine groups.^1^ According to this definition, Sweden’s three largest cities—Stockholm, Malmö, and Gothenburg—and their surrounding municipalities are metropolitan areas. Thus, shorter moves to suburban areas within the metropolitan areas are not defined as counterurban moves in this study. Families moving within or between metropolitan areas are defined as stayers within the metropolitan context. By distinguishing between counterurban moves to medium-sized towns and more rural regions, we aim to add more insights to the literature on the counterurbanisation process in contemporary society.

As migration choices in couples are made at the household level, the residential history of both partners is important. For our analysis, it is hence essential that we have annual data on the household level, with information on both partners’ residential history. While other studies (e.g. Feijten et al., 2008) have shown that previous residential experiences influence migration choices and return moves, to our knowledge no studies have had the possibility to take into account both partners’ residential history, as we do in this study, when analysing return moves. As Feijten et al. (2008) also conclude, their results showing how previous residential experience impacts return migration might be underestimated, as they could only consider whether the migration was a return move for the respondent. Thus, not only are we able to analyse the extent to which there is a ‘return-to-roots’ movement within contemporary counterurbanisation; we are also able to use the information on the place of residence of the parents and parents-in-law to analyse the importance of links to family members at the destination.

From all households in Sweden for the period 2003–2013, we included all couples with young children based on these criteria: both partners aged 25–40; married, registered partners, or cohabiting; have children under the age of 13; and have lived at least three years in one of the three Swedish metropolitan areas.^2^ With these criteria, we strive to exclude migration that is related to family formation or separation. This gives a total of 250,082 young families and 974,628 household-years.

### Methods

Event-history analysis was used to explain the counterurban moves of young families and their relation to place of childhood origin. The first analyses in this paper are based on competing risk, using a discrete-time multinomial logistic regression specification using stcrreg in STATA. This competing-risk regression is based on Fine and Gray’s proportional subdistribution hazard model, which takes into account competing risks when analysing time-to-event outcomes, and observations with the competing event remain in the risk set (Fine & Gray, 1999). The subdistribution hazard ratios (SHRs) obtained from the Fine-Gray model describe which covariates affect the probability of an event occurring over time. Here, we assess how previous residential experiences within families affect the probability of young families making a counterurban move, with the reference category stayers in the metropolitan area. We assess the probability of counterurban moves to medium-sized town areas as the event of interest, with counterurban moves to rural areas as the competing event (Models 2 and 4). We also run competing-risk models with counterurban moves to rural areas as the event of interest in the presence of moves to medium-sized town areas as a competing event (Models 3 and 4). Subsequent analysis was discrete-time binary logistic regression to assess whether family characteristics among counterurban movers originating from a non-metropolitan area are associated with the probability of making a return move.

We start following the household as a family if they fulfil our criteria as described above. For the competing-risk analyses, each family is followed until their first counterurban move, or until 2013 if not migrating. In the discrete-time binary logistic regression, we analyse the probability that a counterurban move is a return move for the group of movers who originate from a non-metropolitan area. Given that return migration requires a previous move, not all are ‘at risk’ of becoming a return mover and it is not self-evident what the group of comparison should be (as discussed by Amcoff & Niedomysl, 2013). In this case, we use onward migrants as comparison; i.e. couples in which at least one of the partners was born outside a metropolitan area and are hence potential return migrants but who choose an alternative counterurban destination. Families in which both partners were born in a metropolitan area are thereby excluded from the analysis of return migration. In all analyses, we stop following families if they separate/divorce, if at least one partner dies, or if at least one partner turns 40. They are then removed from the dataset (censored).

Through these model specifications, we can estimate the probability of a migration event occurring in the current year as a result of the previous year’s characteristics (Allison, 1982, 2014; Yamaguchi, 1991). These models accommodate repeated observations on not only the same household but also time-varying variables from year to year, such as changing family characteristics, which may have an effect on the probability of migrating. The data are organised as a household-year dataset, in which each line represents one year containing information on each household, and the dependent variable indicates whether a move occurs during each year.

The time-varying independent variables representing socioeconomic characteristics within the family are (see also Table 1): highest attained education level, self-employment, source of income each year for both partners, and household disposable income divided into quartiles. Having no source of income from either transfers (parental benefits or student benefits) or employment/self-employment is defined as being not employed. As a standard procedure within event history analysis, time-varying independent variables are lagged by one year as changes in these variables may impact the probability of migrating the following year. We do not lag the variable indicating parental leave, however, as parental benefits are a secure source of income for a dual-income household in the transition associated with moving and can be seen as a facilitating factor that triggers the timing of the move. Demographic variables are the mean age of the partners as well as the number of children in the family under age six and aged six to twelve. We also control for having been born in Sweden (native-born) or not. We also include being self-employed as a covariate, divided by gender. Theoretically, self-employment can make it easier for a family to make a counterurban move, with at least one in the couple being independent of an employer. Case studies have found that lifestyle considerations have led entrepreneurs within the creative and knowledge sectors to move from urban to rural areas with an overrepresentation of female entrepreneurs (e.g. Herslund, 2012), and that rural in-migrants are more often involved in entrepreneurial activities than rural stayers are (e.g. Mitchell & Madden, 2014). Contradictory to these results, register-based studies have found that self-employment prior to a move does not make it more likely for families to leave the metropolitan context (Sandow & Lundholm, 2020), and that rural in-migrants are less often self-employed compared to locals (Eliasson et al., 2015). However, the decision to make a counterurban move can be easier to take for the self-employed if the move is also a return move, as studies (e.g. Audretsch et al., 2012) show that entrepreneurship can be highly dependent on local knowledge and networks.

**Table 1:** Descriptive statistics for all families in the study, based on information on each family during their first year in the study (time t = 0)

Variable	Time period	Mean
Mean age of couple	Time-varying	34
No. of children 0–5 years	Time-varying	1.7
No. of children 6–12 years	Time-varying	1.3
Both in couple highly educated	Time-varying, t-1	35.6%
One in couple highly educated	Time-varying, t-1	27.6%
Household disposable income, SEK^a^	Time-varying, t-1	468,203
Self-employed, woman	Time-varying, t-1	3.8%
Self-employed, man	Time-varying, t-1	8.9%
Not employed, woman	Time-varying, t-1	5.6%
Not employed, man	Time-varying, t-1	5.1%
Parental leave, one in couple	Time-varying	26.9%
Student, one in couple	Time-varying	7.2%
Both spouses native-born	Fixed	68.5%
Place of origin (where at least one in couple lived at age 12):		
Metropolitan area	Fixed	64.4%
Medium-sized town area	Fixed	28.4%
Rural area	Fixed	10.8%
Number of households		250,082

See text in the Methods section for a detailed definition of variables

^a^100,000 SEK is equivalent to approximately 9687 Euro (April 2022)

Descriptive statistics for the independent variables, presented in Table 1, are based on information on each family when we start following them; i.e. when they enter the study. As can be seen, the majority of the households followed in this study are young families in which both partners are Swedish-born, have their origin in a metropolitan area, and are in their mid-30 s when we start following them. About a third are highly educated, and a relatively low share are unemployed. Self-employment is around 9% among fathers and 4% among mothers.

## Results

### The Counterurban Moving Families

A total of 2.6% (6,445) of the young families in this study make a counterurban move as an intact couple during the study period, the majority (60%) of them resettling in medium-sized town areas. As mentioned, the study excludes shorter moves to suburban areas. The majority of the families also make a relatively long move, more than half of them resettling at least 168 km from their previous home in the metropolitan area. Only a small share of the families make a short move, with 10% moving closer than 43 km. Also notable is that the distance is measured in Euclidean distance, meaning that the travelling distance is even longer.

### Impact of Childhood Origin on Destination When Making A Counterurban Move

It is known that previous residential experience, for example during childhood, influences one’s residential choices later in life. The majority of the counterurban movers also had their childhood outside a metropolitan area. In 75% of all counterurban families, at least one of the partners is not originally from a metropolitan area; the corresponding number is 40% for the families not moving from the metropolitan area. To further examine the impact of childhood origin on making a counterurban move and the type of destination families resettle in, we ran competing-risk regressions with all the families in the study (see Models 1 and 3 in Table 2). The results clearly show that childhood origin impacts the likelihood of making a counterurban move. Families in which one or both partners had their childhood outside a metropolitan area are significantly more likely to make a counterurban move compared to those who grew up in a metropolitan area. Childhood origin also impacts the choice of destination: Growing up in a rural area makes a counterurban move to a rural area compared to a town area more than five times more likely (Model 3). It is three times more likely for a family to move to a destination in a medium-sized town area if one or both partners had their childhood in a similar type of region (Model 1). Growing up in a rural area also increases the likelihood of becoming a counterurban mover to a medium-sized town (Model 1). Overall, this indicates the importance of experience of previous residence for choice of destination. This might reflect that the experiences from childhood residence have created a preference for moving to a similar residential environment later in life when having children of one’s own. But it could also be the expected result of counterurban moves to the actual place of origin, meaning that the counterurban move is actually a return move.

**Table 2:** Results from competing-risk models of out-migration from metropolitan area to medium-sized town area and competing-risk models of out-migration from metropolitan area to rural area

	Model 1 Out-migration to medium-sized town area^a^	Model 2 Out-migration to medium-sized town area^a^ (return moves not included in model)	Model 3 Out-migration to rural area^b^	Model 4 Out-migration to rural area^b^ (return moves not included in model)
Variables	SHR	SHR	SHR	SHR
Mean age of couple	0.97***	0.97***	0.98**	0.99
No. of children 0–5 years	1.12***	1.07	1.15***	1.11*
No. of children 6–12 years	1.00	1.01	1.09**	1.10
Both in couple highly educated	1.37***	1.27***	1.16**	1.21**
One in couple highly educated	1.12*	0.99	1.07	0.98
Household disposable income, quartiles (< 25% ref.):				
25–49%	0.81***	0.76***	0.75***	0.68***
50–74%	0.65***	0.57***	0.52***	0.39***
75–100%	0.51***	0.45***	0.42***	0.33***
Self-employed, woman	1.15	1.37***	0.97	0.97
Self-employed, man	0.82***	0.80**	1.09	1.06
Not employed, woman	1.04	0.99	1.03	1.03
Not employed, man	0.92	1.02	1.00	1.00
Parental leave, one in couple	1.34***	1.32***	1.28***	1.22**
Student, one in couple	0.99	1.12	0.85	0.91
Both spouses native-born	1.52***	1.26***	1.90***	1.45***
Place of origin in metropolitan area (where at least one in couple lived at age 12)	0.50***	0.65***	0.53***	0.86*
Place of origin in rural area, (where at least one in couple lived at age 12)	1.22***	1.90***	5.25***	2.16***
Place of origin in medium-sized town (where at least one in couple lived at age 12)	3.60***	1.98***	0.73***	1.22**
Time period of the move (2004–2006 ref.):				
2007–2009	1.04	0.98	0.84**	0.81**
2010–2013	0.98	0.87**	0.83***	0.70***
Log pseudolikelihood	-43,159.78	-28,689.98	-28,330.20	-16,983.42
N household-years (households)	974,296 (250,081)	966,531 (247,704)	974,315 (250,081)	966,547 (247,704)

*p<0.05; **p<0.01; ***p<0.001

*a*: In the equations for out-migrating to a medium-sized town area versus not moving, out-migrating to a rural area is treated as a competing risk

*b*: In the equations for out-migrating to a rural area versus not moving, out-migrating to a medium-sized town area is treated as a competing risk

SHR: Subdistribution Hazard Ratio

To examine whether childhood origin has an effect on counterurban moves beyond return migration, we ran competing-risk models without the families returning to their place of origin (see Models 2 and 4 in Table 2). Overall, and in line with the results for all counterurban moves, previous experience of residence outside a metropolitan area increases the likelihood of making a non-return counterurban move. The size of the estimated intensities related to childhood origin is lower, as expected, but are still statistically significant. When return moves are excluded, we see that origin in both a town and a rural area means a greater likelihood of being a counterurban mover, to both rural and town destinations. Thus, childhood environment is associated with destination choice even when a move is not related to links at the destination. Still, the association between destination choice and previous residential experiences is the strongest for counterurban moves to rural areas.

In line with a previous study by Sandow and Lundholm (2020), the characteristics shared by all the counterurban moving families compared to stayers are that they are more likely to be Swedish-born, on parental leave, highly educated, but not high-income earners, regardless of destination. The migration intensities increase with number of preschool children and decrease with age. A possible interpretation of this could be that counterurban migrants have often migrated to metropolitan areas for higher education, lived there for several years, formed a family, and then, decided to move to another kind of residential context than the big city. The results also indicate that they choose to do so when their family is growing and when one of the partners is on parental leave. Regarding self-employment, the results are more complex. Overall, we find that self-employment does not increase counterurban migration intensities, with one exception. Migration intensities to medium-sized town areas increase if the mother is self-employed prior to the move and the move is not a return move (Model 2). Further studies are needed in order to investigate the interrelation between gender and self-employment in counterurban migration. Furthermore, the estimated subdistribution hazard ratios (SHR) in Table 2 indicate that counterurban migration intensities have decreased slightly in the later time period, even those not related to return migration.

### Counterurbanisation and ‘Return-to-Roots’

**Fig. 1:**
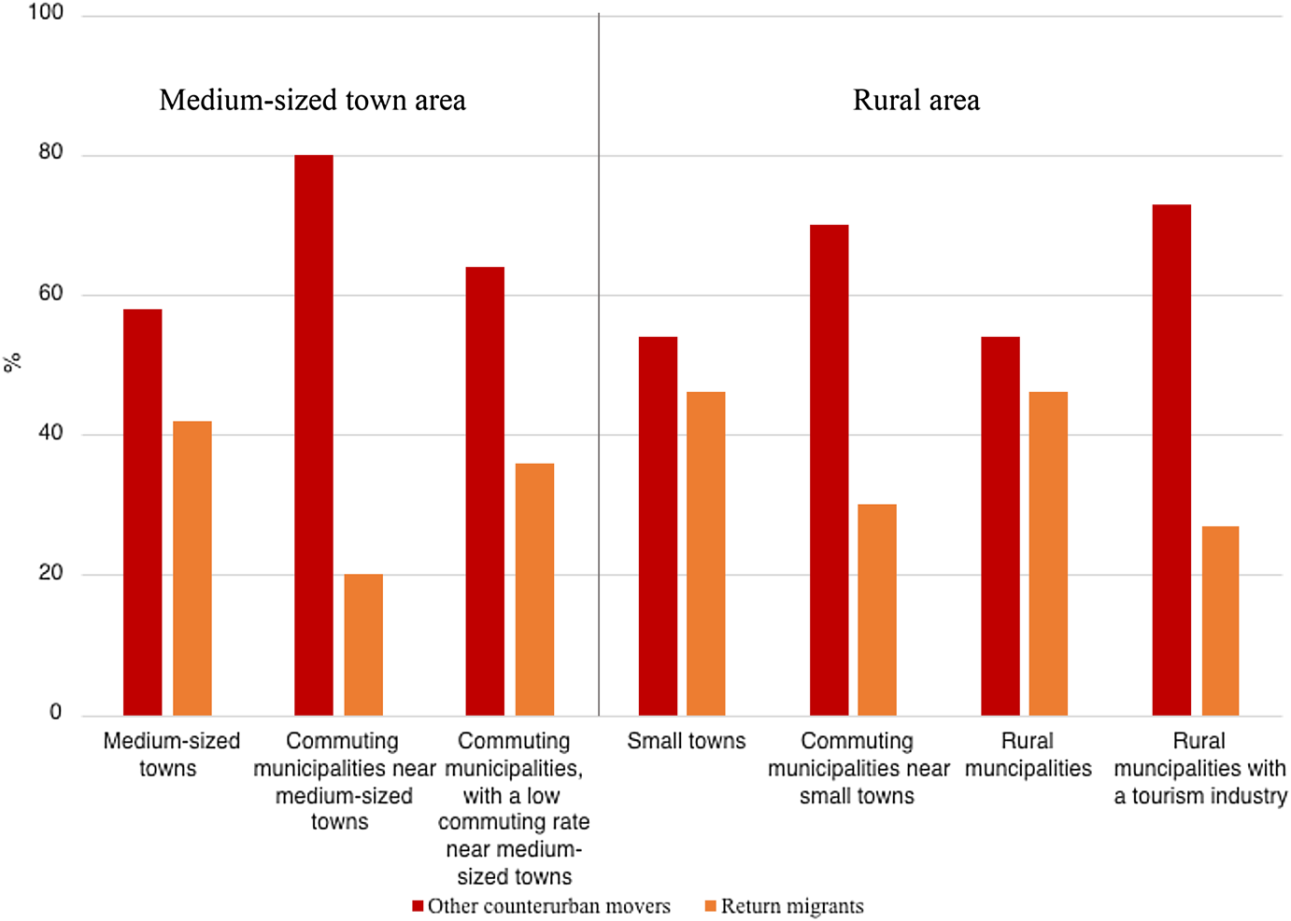
Share of return migrants among counterurban moving families to each destination type

Among all young families making a counterurban move in Sweden, we see a clear pattern of a ‘return-to-roots’ movement; i.e. returning to one’s origin and/or family. In total, of all counterurban families, 37% move to the area where one or both of the partners grew up, a so-called return move. As can be seen in Table 3, the counterurban moving families that return to one of the partners’ places of childhood origin are, to a higher extent than other counterurban moving families, highly educated and in the highest income quartile the year before the move. Among the other counterurban moving families, it is more common for one of the partners to be enrolled in studies or not in employment and for the mother to be self-employed the year prior to the move. A higher share of these non-return-related counterurban moving families is also non-native with family roots in a metropolitan area. Figure 1 shows the destination categories for all counterurban moving families, divided into those making a return move and moves not related to return migration. Overall, the share of return migrants is highest in the rural areas: the small towns and rural municipalities. The commuting settlements and rural areas characterised by the tourism industry have a higher share of counterurban movers not related to return migration. Preferences among these other counterurban movers seem to typically direct them towards either suburbanisation and commuting or attractive rural areas. The non-return-related counterurban movers also move shorter distances on average, and a larger share of them move less than 50 km, compared to moves related to return migration (see Table 3). This is understandable, as non-return migrants potentially have more destinations to choose from and can opt for a short-distance choice. It could also involve a spillover of suburban movers who accept longer commutes from the metropolitan hinterland.

**Table 3:** Characteristics of counterurban moving families at the time of the move, divided based on whether the move is related to return migration or not

Variables	Counterurban moves	
	Not related to return migration	Return migration
Mean age of couple	34.8	34.7
No. of children 0–5 years	1.3	1.6
No. of children 6–12 years	0.7	0.5
Both in couple highly educated	41.4%	54.7%
One in couple highly educated	24.3%	26.1%
*Household disposable income, quartiles:*		
0–25%	33.5%	21.1%
25–49%	27.6%	26.7%
50–74%	20.3%	28.1%
75–100%	18.7%	25.1%
Self-employed, woman	5.7%	3.9%
Self-employed, man	9.8%	9.0%
Not employed, woman	5.7%	2.7%
Not employed, man	4.8%	1.7%
Parental leave, one in couple	24.0%	25.2%
Student, one in couple	7.9%	4.2%
Both spouses native-born	71.4%	87.7%
*Place of origin (where at least one in couple lived at age 12):*		
Metropolitan area	54.4%	36.2%
Medium-sized town area	40.6%	62.3%
Rural area	17.4%	30.4%
*Migration distance:*		
< 50 km	18.0%	6.4%
51–100 km	23.6%	17.6%
101–200 km	20.9%	27.4%
201–300 km	14.5%	18.9%
301 + km	23.1%	29.7%
Mean	200 km	250 km
Median	144 km	195 km
Number of households	4,014	2,371

Return migration and moving towards family members seem to be intertwined. Nearly all families making a return move to one of the partners’ childhood municipalities have parents/parents-in-law still living there (94%). This indicates that family bonds are important when moving. Moving closer to family networks has also been found to be an important motive for counterurban migration (Haartsen & Thissen, 2014; Scott et al., 2017). While moving to live closer to one’s family network often coincides with moving back to one’s childhood area, this is not always the case. For instance, one’s parents may have moved after the children left the parental home, or one may be non-native. We also find that among families of non-native background, a third of them move to the same areas as their parents when resettling in a non-metropolitan area. This further strengthens the results, showing that closeness to relatives is important when choosing a destination outside a metropolitan area. In line with the general pattern of return migration in Sweden (Amcoff & Niedomysl, 2015; Statistics Sweden, 2017), it is also more common for the young families to move to the place where only the woman’s parents live than to where only the man’s parents live.

In order to assess differences in family characteristics between return and non-return migrants within the counterurban group, we performed a discrete-time binary logistic regression (Table 4). Here, only counterurban movers among whom one or both partners grew up outside a metropolitan area, and thus, potentially can choose to make a return move, are included in the analysis. The results indicate lower probabilities that the counterurban move will be a return move if one of the partners was born in a metropolitan area and higher probabilities among high-income earners and native-born and if there are preschool children in the family. Hence, there is no evidence that ‘failed migration’ would be typical when it comes to this group of return migrants, as return migrants are not more likely to be unemployed compared to other counterurban movers and are less likely to belong to the lowest income category. It is also less likely for the counterurban move to be a return move if the mother is self-employed prior to the family’s decision to relocate outside the metropolitan context. This result is somewhat unexpected, as return moves are more often towards the female’s place of childhood origin and one could expect local networks to be in favour of female self-employment when making a return move. There seems to be a tendency that counterurban moves are more likely to be return migration in the later period compared to the earlier one.

**Table 4:** Out-migration from metropolitan areas: discrete-time logistic regression of return migration among counterurban movers originating from a non-metropolitan area (1 = return mover; 0 = other counterurban mover)

Variables	Odds Ratio
Mean age of couple	0.97**
No. of children 0–5 years	1.18***
No. of children 6–12 years	0.97
Both in couple highly educated	1.06
One in couple highly educated	1.24*
*Household disposable income, quartiles (*< *25% ref.):*	
25–49%	1.35***
50–74%	1.71***
75–100%	1.65***
Self-employed, woman	0.63***
Self-employed, man	1.05
Not employed, woman	0.97
Not employed, man	0.82
Parental leave, one in couple	1.06
Student, one in couple	0.79
Both spouses native-born	2.57***
Place of origin in metropolitan area (where at least one in couple lived at age 12)	0.61***
Place of origin in rural area (where at least one in couple lived at age 12)	1.43***
Place of origin in medium-sized town (where at least one in couple lived at age 12)	1.48***
*Time period of move (2004–2006 ref.)*	
2007–2009	1.07
2010–2013	1.22**
Constant	0.36**
N (Households)	5,366

^*^_p<0.05; **p<0.01; ***p<0.001_

## Concluding Discussion

Drawing on evidence from Sweden, this paper offers a broader perspective on counterurban moves by nuancing the idea that counterurbanisation is foremost a phenomenon involving urbanites wanting to start a new life in an idyllic rural setting. Thus, this approach recognises recent thinking in counterurbanisation research (Haartsen & Thissen, 2014; Scott et al., 2017), acknowledging the role of family members and family roots at the destination, drawing on a life course perspective on migration choices. Through register data on all young families with children living in metropolitan areas, we have been able to examine the extent to which counterurban migration is a return migration phenomenon in contemporary Sweden. The data have allowed us to take into account both partners’ childhood residential histories, which to our knowledge has not been done previously.

By distinguishing between destination types, this study shows that the direction of counterurban moves from metropolitan regions is not mainly to more rural areas but rather largely to medium-sized town areas. To find the reasons behind this, and determine whether it is a long-term trend, require further studies in which diverging tendencies for different counterurban destinations are investigated. From the results here, we can conclude that contemporary counterurban migration in Sweden is strongly associated with the previous residential environment, even if it is not necessarily the place of origin. Similar to Feijten et al. (2008), we found that especially those with roots in rural areas are more inclined to become counterurban movers and also to choose a rural destination, even if it is not their place of origin.

We found that the young families with children moving out of metropolitan areas in Sweden are likely to be younger, in employment, highly educated, have preschool children, and are more likely to be on parental leave, compared to families choosing to stay in the metropolitan areas. While the general counterurban moves of the mid-1980s to the mid-1990s in Sweden (Lindgren, 2003) tended to be triggered by unemployment events, this is not the case for the counterurban families in focus in this study. An interesting finding, however, is that the counterurban movers are more likely to belong to the lowest income category, but are also more likely to be highly educated, compared to stayers. This could reflect that they are displaced by high property prices. To explain the result that families with a self-employed female to a greater extent choose medium-sized town areas to which the family has no previous residential connection, further studies are required. It would also be of interest to focus on the employment situation after the move and explore how career paths develop, the extent to which the counterurban movers become entrepreneurs, and possible gender differences.

The results show that three quarters of the counterurban moves consist of urban residents with a background in other parts of the country. When it comes to actual return to place of childhood origin, nearly four out of ten of all young counterurban moving families are making a return move. As the destination is often both the place of origin and where close relatives live, return motives and family reasons are presumably intertwined for these families when leaving the metropolitan area. Nevertheless, the results provide support for including a life course perspective in understanding counterurban migration and return migration, acknowledging the importance of linked lives. One interpretation of the results is that proximity to parents, parents-in-law, or other relatives can be important for young families with children, and that this can promote a move. For young families with children, moving closer to grandparents allows the children to get to know them better, and they can also assist with homework and transportation to and from school and other activities. The need for support can also be in the other direction, with the younger generation returning to provide care to the older generation. The tendency we see of young families moving closer to the woman’s place of origin and roots may indicate a ‘grandmother effect’ whereby maternal grandparents are particularly important in providing support, as discussed by Gosh et al. (2019).

This study contributes to the literature following, e.g. von Reichert et al. (2014), by demonstrating that there is more to return migration than the success-failure dichotomy. Compared to other counterurban movers, return migrants seem to be better off economically. This is in contrast to other studies including all return migrants, in which unemployment or income loss has been found to trigger return migration (Amcoff & Niedomysl, 2013). In this study focusing on young families moving out of metropolitan areas, we find that family members and roots at the destination, rather than economic necessities, seem to be the main drivers of return migration. The findings that return migration constitutes a substantial part of contemporary counterurban migration flow, and that the likelihood that the move is a return move have increased in the latter time period, call for further studies.

From a policy perspective, the results suggest that potential counterurban migrants are often found among former out-migrants, especially those with families remaining in the region. This is especially true for those moving to small towns and rural areas, where the share of returning families among in-migrants is the highest. In line with the study by Bijker and Haartsen (2012), we also find that the counterurban moves that are not related to return migration to a greater extent involve a choice of more attractive rural areas (i.e. within commuting distance to bigger cities or with a tourism industry profile). Future qualitative studies could contribute to a deeper contextual understanding of geographical preferences in young families’ destination choices.

Although the phenomenon of counterurbanisation was relatively small during the period of this study (2003–2013), the national statistics indicate a continuous increase in young families leaving the metropolitan areas (Statistics Sweden, 2021). It would therefore be of interest to further explore whether this migration behaviour is about to change the general internal migration pattern, not least in relation to the COVID-19 pandemic. In the post-pandemic future, changing preferences for ‘hybrid working’, combining remote work with office work, along with employers increasingly offering the possibility for remote work, might facilitate an accelerating out-migration from big cities to smaller cities or rural areas and enhance return migration. Also, the results from this study call for further investigation of different groups of return and counterurban migrants, as we see that return migration in this group diverges from the general pattern when it comes to socioeconomic characteristics. While this study has focused solely on young families moving beyond the metropolitan hinterland, future studies could also focus on families moving shorter distances and on those choosing rural areas closer to the metropolitan hinterland.

## Data Availability

Data cannot be shared publicly because of ethical and data safety reasons. Data are available from Statistics Sweden after approval of the research project by an Ethical Committee and by the data safety committees of the Swedish Authorities for researchers who meet the criteria for access to confidential data. The database used in this study was constructed after approval from the Ethical Committee in Sweden (https://etikprovningsmyndigheten.se/) and from the data safety committees of Statistics Sweden (https://www.scb.se/en/).

## Footnotes

1 The categories within ‘medium-sized town area’ are medium-sized towns, commuting municipalities near medium-sized towns, and commuting municipalities with a low commuting rate near medium-sized towns. The categories within ‘rural area’ are small towns, commuting municipalities near small towns, rural municipalities, and rural municipalities with a tourism profile industry (SKR 2016).

2 For all families we start following in 2003, we go back to 2001 in the data to control for their having lived in the metropolitan area for at least three years.

## Code Availability

Available by authors by request.
